# Inferring Pairwise Interactions from Biological Data Using Maximum-Entropy Probability Models

**DOI:** 10.1371/journal.pcbi.1004182

**Published:** 2015-07-30

**Authors:** Richard R. Stein, Debora S. Marks, Chris Sander

**Affiliations:** 1 Computational Biology Program, Sloan Kettering Institute, Memorial Sloan Kettering Cancer Center, New York, New York, United States of America; 2 Department of Systems Biology, Harvard Medical School, Boston, Massachusetts, United States of America; University of Missouri, UNITED STATES

## Abstract

Maximum entropy-based inference methods have been successfully used to infer direct interactions from biological datasets such as gene expression data or sequence ensembles. Here, we review undirected pairwise maximum-entropy probability models in two categories of data types, those with continuous and categorical random variables. As a concrete example, we present recently developed inference methods from the field of protein contact prediction and show that a basic set of assumptions leads to similar solution strategies for inferring the model parameters in both variable types. These parameters reflect interactive couplings between observables, which can be used to predict global properties of the biological system. Such methods are applicable to the important problems of protein 3-D structure prediction and association of gene–gene networks, and they enable potential applications to the analysis of gene alteration patterns and to protein design.

## Introduction

Modern high-throughput techniques allow for the quantitative analysis of various components of the cell. This ability opens the door to analyzing and understanding complex interaction patterns of cellular regulation, organization, and evolution. In the last few years, **undirected pairwise maximum-entropy probability models** have been introduced to analyze biological data and have performed well, disentangling **direct interactions** from artifacts introduced by intermediates or spurious coupling effects. Their performance has been studied for diverse problems, such as gene network inference [1,2], analysis of neural populations [3,4], protein contact prediction [5–8], analysis of a text corpus [9], modeling of animal flocks [10], and prediction of multidrug effects [11]. Statistical inference methods using partial correlations in the context of graphical Gaussian models (GGMs) have led to similar results and provide a more intuitive understanding of direct versus indirect interactions by employing the concept of conditional independence [12,13].

Our goal here is to derive a unified framework for pairwise maximum-entropy probability models for continuous and categorical variables and to discuss some of the recent inference approaches presented in the field of protein contact prediction. The structure of the manuscript is as follows: (1) introduction and statement of the problem, (2) deriving the probabilistic model, (3) inference of interactions, (4) scoring functions for the pairwise interaction strengths, and (5) discussion of results, improvements and applications.

Better knowledge of these methods, along with links to existing implementations in terms of software packages, may be helpful to improve the quality of biological data analysis compared to standard correlation-based methods and increase our ability to make predictions of interactions that define the properties of a biological system. In the following, we highlight the power of inference methods based on the maximum-entropy assumption using two examples of biological problems: inferring networks from gene expression data and residue contacts in proteins from multiple sequence alignments. We compare solutions obtained using (1) correlation-based inference and (2) inference based on pairwise maximum-entropy probability models (or their incarnation in the continuous case, the multivariate Gaussian distribution).

### Gene association networks

Pairwise associations between genes and proteins can be determined by a variety of data types, such as gene expression or protein abundance. Association between entities in these data types are commonly estimated by the sample **Pearson correlation** coefficient computed for each pair of variables *x*_*i*_ and *x*_*j*_ from the set of random variables *x*_*1*_,…, *x*_*L*_. In particular, for *M* given samples in *L* measured variables, $x^{1}={\left(x_{1}^{1},\ldots ,x_{L}^{1}\right)}^{\text{T}},\ldots ,x^{M}={\left(x_{1}^{M},\ldots ,x_{L}^{M}\right)}^{\text{T}}\in {\mathbb{R}}^{L}$, it is defined as,

$$r_{ij}:=\frac{{\hat{C}}_{ij}}{\sqrt{{\hat{C}}_{ii}{\hat{C}}_{jj}}},$$

where ${\hat{C}}_{ij}:=\frac{1}{M}\sum_{m=1}^{M}(x_{i}^{m}-\bar{x_{i}})(x_{j}^{m}-\bar{x_{j}})$ denotes the (*i*, *j*)-element of the empirical covariance matrix $\hat{C}={\left({\hat{C}}_{ij}\right)}_{i,j=1,\ldots ,L}$. The sample mean operator $\bar{\;\cdot \;}$ provides the empirical mean from the measured data and is defined as $\bar{x_{i}}:=\frac{1}{M}\sum_{m=1}^{M}x_{i}^{m}$. A simple way to characterize dependencies in data is to classify two variables as being dependent if the absolute value of their correlation coefficient is above a certain threshold (and independent otherwise) and then use those pairs to draw a so-called relevance network [14]. However, the Pearson correlation is a misleading measure for direct dependence as it only reflects the association between two variables while ignoring the influence of the remaining ones. Therefore, the relevance network approach is not suitable to deduce direct interactions from a dataset [15–18]. The **partial correlation** between two variables removes the variational effect due to the influence of the remaining variables (Cramér [19], p. 306). To illustrate this, let’s take a simplified example with three random variables *x*_A_, *x*_B_, *x*_C_. Without loss of generality, we can scale each of these variables to zero-mean and unit-standard deviation by $x_{i}\mapsto \left(x_{i}-\bar{x_{i}}\right)/\sqrt{{\hat{C}}_{ii}}$, which simplifies the correlation coefficient to $r_{ij}\equiv \bar{x_{i}x_{j}}$. The sample partial correlation coefficient of a three-variable system between *x*_A_ and *x*_B_ *given x*_C_ is then defined as [19,20]

$$r_{\text{AB}\cdot \text{C}}=\frac{r_{\text{AB}}-r_{\text{BC}}r_{\text{AC}}}{\sqrt{1-r_{\text{AC}}^{2}}\sqrt{1-r_{\text{BC}}^{2}}}\equiv -\frac{{\left({\hat{C}}^{-1}\right)}_{\text{AB}}}{\sqrt{{\left({\hat{C}}^{-1}\right)}_{\text{AA}}{\left({\hat{C}}^{-1}\right)}_{\text{BB}}}}.$$

**Fig 1:**
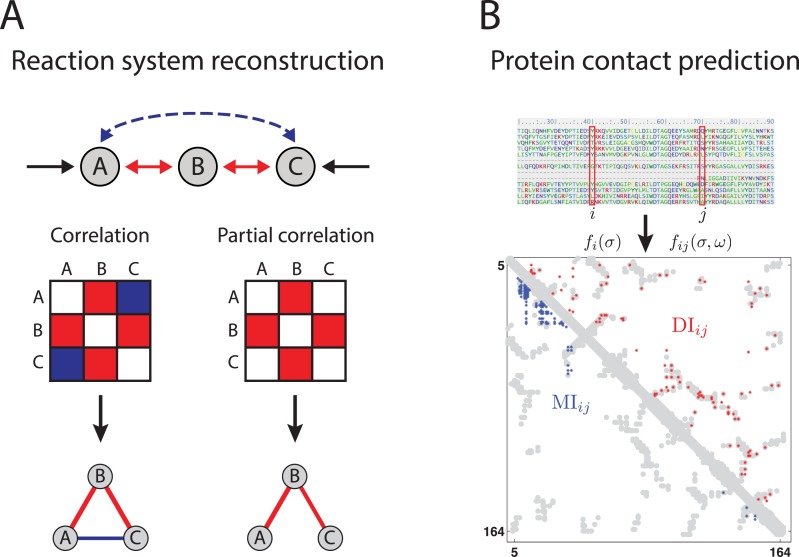
Reaction system reconstruction and protein contact prediction. Association results of correlation-based and maximum-entropy methods on biological data from an *in silico* reaction system (A) and protein contacts (B). (A) Analysis by Pearson’s correlation yields interactions associating all three compounds A, B, and C, in contrast to the partial correlation approach which omits the “false” link between A and C. (Fig 1A based on [21].) (B) Protein contact prediction for the human RAS protein using the correlation-based mutual information, MI, and the maximum-entropy based direct information, DI, (blue and red, respectively). The 150 highest scoring contacts from both methods are plotted on the protein contacts from experimentally determined structure in gray. (Fig 1B based on [6].)

The latter equivalence by Cramer’s rule holds if the empirical covariance matrix, $\hat{C}={\left({\hat{C}}_{ij}\right)}_{i,j\in \left\{\text{A},\text{B},\text{C}\right\}}$, is invertible. Krumsiek et al. [21] studied the Pearson correlations and partial correlations in data generated by an *in silico* reaction system consisting of three components A, B, C with reactions between A and B, and B and C (Fig 1A). A graphical comparison of Pearson’s correlations, *r*_AB_, *r*_AC_ *r*_BC_, versus the corresponding partial correlations, *r*_AB·C_, *r*_AC·B_, *r*_BC·A_, shows that variables A and C appear to be correlated when using Pearson’s correlation as a dependency measure since both are highly correlated with variable B, which results in a false inferred reaction *r*_AC_. The strength of the incorrectly inferred interaction can be numerically large and therefore particularly misleading if there are multiple intermediate variables B [22]. The partial correlation analysis removes the effect of the mediating variable(s) B and correctly recovers the underlying interaction structure. This is always true for variables following a multivariate Gaussian distribution, but also seems to work empirically on realistic systems as Krumsiek et al. [21] have shown for more complex reaction structures than the example presented here.

### Protein contact prediction

The idea that protein contacts can be extracted from the evolutionary family record was formulated and tested some time ago [23–26]. The principle used here is that slightly deleterious mutations are compensated during evolution by mutations of residues in contact in order to maintain the function and, by implication, the shape of the protein. Protein residues that are close in space in the folded protein are often mutated in a correlated manner. The main problem here is that one has to disentangle the directly co-evolving residues and remove transitive correlations from the large number of other co-variations in protein sequences that arise due to statistical noise or phylogenetic sampling bias in the sequence family. Interactions not internal to the protein are, for example, evolutionary constraints on residues involved in oligomerization, protein–protein, protein–substrate interactions [6,27,28]. In particular, the empirical single-site and pair frequency counts in residue *i* and in residues *i* and *j* for elements *σ*, *ω* of the 20-element amino acid alphabet plus gap, *f*_*i*_(*σ*) and *f*_*ij*_(*σ*, *ω*), are extracted from a representative multiple sequence alignment under applied reweighting to account for biases due to undersampling. Correlated evolution in these positions was analyzed, e.g., by [29], by using the **mutual information** between residue *i* and *j*,

$${\text{MI}}_{ij}=\underset{\sigma ,\omega }{\sum }f_{ij}(\sigma ,\omega )\text{ln}\left(\frac{f_{ij}(\sigma ,\omega )}{f_{i}(\sigma )f_{j}(\omega )}\right).$$

Although results did show promise, an important improvement was made years later by using a maximum-entropy approach on the same setup [5–7,30]. In this framework, the **direct information** of residue *i* and *j* was introduced by replacing *f*_*ij*_ in the mutual information by $P_{ij}^{\text{dir}}$,

$${\text{DI}}_{ij}=\sum_{\sigma ,\omega }P_{ij}^{\text{dir}}(\sigma ,\omega )\text{ln}\left(\frac{P_{ij}^{\text{dir}}(\sigma ,\omega )}{f_{i}(\sigma )f_{j}(\omega )}\right),$$ (1)

where $P_{ij}^{\text{dir}}(\sigma ,\omega )=\frac{1}{Z_{ij}}\text{exp}\left(e_{ij}(\sigma ,\omega )+{\tilde{h}}_{i}(\sigma )+{\tilde{h}}_{j}(\omega )\right)$ and ${\tilde{h}}_{i}(\sigma ),{\tilde{h}}_{j}(\omega )$ and *Z*_*ij*_ are chosen such that $P_{ij}^{\text{dir}}$, which is based on a pairwise probability model of an amino acid sequence compatible with the iso-structural sequence family, is consistent with the single-site frequency counts. In an approximative solution, [6,7] determined the contact strength between the amino acids *σ* and *ω* in position *i* and *j*, respectively, by

$$e_{ij}(\sigma ,\omega )≃-{\left(C^{-1}(\sigma ,\omega )\right)}_{ij}.$$ (2)

Here, (*C* ^−1^(*σ*,*ω*))_*ij*_ denotes the inverse element corresponding to *C*_*ij*_ (*σ*,*ω*) ≡ *f*_*ij*_(*σ*,*ω*) − *f*_*i*_(*σ*) *f*_*j*_(*ω*) for amino acids *σ*, *ω* from a subset of 20 out of the 21 different states (the so-called gauge fixing, see below). The comparison of contact prediction results based on MI- and DI-score for the RAS human protein on top of the actual crystal structure shows a much more accurate prediction result when using the direct information instead of the mutual information (Fig 1B).

The next section lays the foundation to deriving maximum-entropy models for the two data types: continuous, as used in the first example, and categorical, as used in the second one. Subsequently, we will present inference techniques to solve for their interaction parameters.

## Deriving the Probabilistic Model

Ideally, one would like to use a probabilistic model that is, on the one hand, able to capture all orders of directed interactions of all observables at play and, on the other hand, correctly reproduces the observed and to-be-predicted frequencies. However, this would require a prohibitively large number of observed data points. For this reason, we restrict ourselves to probabilistic models with terms up to second order, which we derive for continuous, real-valued variables, and extend this framework to models with categorical variables that are suitable, for example, to treat sequence information in the next section.

### Model formulation for continuous random variables

We model the occurrence of sets of events in a particular biological system by a multivariate probability distribution *P*(**x**) of *L* random variables **x** = (*x*_1_,…, *x*_*L*_)^T^ ∈ℝ^*L*^ that is, on the one hand, consistent with the mean and covariance obtained from *M* observed data values **x** ^1^,…, **x** ^*M*^ and, on the other hand, maximizing the information entropy, *S*, to obtain the simplest possible probability model consistent with the data. At this point, each of the data’s variables *x*_*i*_ is continuously distributed on real values. In a biological example, these data originate from gene expression studies and each variable *x*_*i*_ corresponds to the normalized mRNA level of a gene measured in *M* samples. As an example, a recent pan-cancer study of The Cancer Genome Atlas (TCGA) provided mRNA levels from *M* = 3,299 patient tumor samples from 12 cancer types [31]. The problem can be large, e.g., in the case of a gene–gene association study one has *L* ≈ 20,000 human genes.

The first constraint on the unknown probability distribution, *P*: ℝ^*L*^ →ℝ_≥0_ is that its integral normalizes to 1,

$$\int_{x}P(x)\text{ d}x=1,$$ (3)

which is a natural requirement on any probability distribution. Additionally, the first moment of variable *x*_*i*_ is supposed to match the value of the corresponding sample mean over *M* measurements in each *i* = 1,…, *L*,

$$\langle x_{i}\rangle =\int_{x}P(x)x_{i}\text{ d}x=\frac{1}{M}\sum_{m=1}^{M}x_{i}^{m}=\bar{x_{i}},$$ (4)

where we define the *n*-th moment of the random variable *x*_*i*_ distributed by the multivariate probability distribution *P* as $\langle x_{i}^{n}\rangle :=\int_{x}P(x)x_{i}^{n}\text{ d}x$. Analogously, the second moment of the variables *x*_*i*_ and *x*_*j*_ and its corresponding empirical expectation is supposed to be equal,

$$\langle x_{i}x_{j}\rangle =\int_{x}P(x)x_{i}x_{j}\text{ d}x=\frac{1}{M}\sum_{m=1}^{M}x_{i}^{m}x_{j}^{m}=\bar{x_{i}x_{j}}$$ (5)

for *i*, *j* = 1,…, *L*. Taken together, Eqs 4 and 5 constrain the distribution’s covariance matrix to be coherent to the empirical covariance matrix. Finally, the probability distribution should maximize the information entropy,

$$\text{maximize }S=-\int_{x}P(x)\text{ln }P(x)\text{ d}x$$ (6)

with the natural logarithm ln. A well-known analytical strategy to find functional extrema under equality constraints is the **method of Lagrange multipliers** [32], which converts a constrained optimization problem into an unconstrained one by means of the Lagrangian ℒ. In our case, the probability distribution maximizing the entropy (Eq 6) subject to Eqs 3–5 is found as the stationary point of the Lagrangian ℒ=ℒ(P(x);α,β,γ) [33,34],

$$ℒ=S+\alpha \left(\langle 1\rangle -1\right)+\sum_{i=1}^{L}\beta_{i}\left(\langle x_{i}\rangle -\bar{x_{i}}\right)+\sum_{i,j=1}^{L}\gamma_{ij}\left(\langle x_{i}x_{j}\rangle -\bar{x_{i}x_{j}}\right).$$ (7)

The real-valued Lagrange multipliers *α*, ***β*** = (*β*_*i*_)_*i* = 1,…, *L*_ and ***γ*** = (*γ*_*ij*_)_*i,j* = 1,…, *L*_ correspond to the constraints Eqs 3, 4, and 5, respectively. The maximizing probability distribution is then found by setting the functional derivative of ℒ with respect to the unknown density *P*(**x**) to zero [33,35],

$$\frac{\delta ℒ}{\delta P(x)}=0\;⟹\;-\text{ln }P(x)-1+\alpha +\sum_{i=1}^{L}\beta_{i}x_{i}+\sum_{i,j=1}^{L}\gamma_{ij}x_{i}x_{j}=0.$$

Its solution is the **pairwise maximum-entropy probability distribution**,

$$P(x;\beta ,\gamma )=\text{exp}\left(-1+\alpha +\sum_{i=1}^{L}\beta_{i}x_{i}+\sum_{i,j=1}^{L}\gamma_{ij}x_{i}x_{j}\right)=\frac{1}{Z}{\text{e}}^{-ℋ(x;\beta ,\gamma )}$$ (8)

which is contained in the family of exponential probability distributions and assigns a non-negative probability to any system configuration **x** = (*x*_1_,…, *x*_*L*_)^T^ ∈ℝ^*L*^. For the second identity, we introduced the **partition function** as normalization constant,

$$Z(\beta ,\gamma ):=\int_{x}\text{exp}\left(\sum_{i=1}^{L}\beta_{i}x_{i}+\sum_{i,j=1}^{L}\gamma_{ij}x_{i}x_{j}\right)\text{ d}x\equiv \text{exp}\left(1-\alpha \right)$$

with the Hamiltonian,$ℋ(x):=-\sum_{i=1}^{L}\beta_{i}x_{i}-\sum_{i,j=1}^{L}\gamma_{ij}x_{i}x_{j}$. It can be shown by means of the information inequality that Eq 8 is the unique maximum-entropy distribution satisfying the constraints Eqs 3–5 (Cover and Thomas [35], p. 410). Note that *α* is fully determined for given ***β*** = (*β*_*i*_ *)* and ***γ*** = (*γ*_*ij*_) by the normalization constraint Eq 3 and is therefore not a free parameter. The right-hand representation of Eq 8 is also referred to as **Boltzmann distribution**. The matrix of Lagrange multipliers ***γ*** = (*γ*_*ij*_) has to have full rank in order to ensure a unique parametrization of *P*(**x**), otherwise, one can eliminate dependent constraints [33,36]. In addition, for the integrals in Eqs 3–6 to converge with respect to *L*-dimensional Lebesgue measure, we require ***γ*** to be negative definite, i.e., all of its eigenvalues to be negative or $\sum_{i,j}\gamma_{ij}x_{i}x_{j}=x^{\text{T}}\gamma x<0$ for **x** ≠ **0**.

### Concept of entropy maximization

Shannon states in his seminal work that information and (information) entropy are linked: the more information is encoded in the system, the lower its entropy [37]. Jaynes introduced the entropy maximization principle, which selects for the probability distribution that is (1) in agreement with the measured constraints and (2) contains the least information about the probability distribution [38–40]. In particular, any unnecessary information would lower the entropy and, thus, introduce biases and allow overfitting. As demonstrated in the section above, the assumption of entropy maximization under first and second moment constraints results in an exponential model or Markov random field (in log-linear form) and many of the properties shown here can be generalized to this model class [41]. On the other hand, there is some analogy of entropy as introduced by Shannon to the thermodynamic notion of entropy. Here, the Second law of Thermodynamics states that each isolated system monotonically evolves in time towards a state of maximum entropy, the equilibrium. A thorough discussion of this analogy and its limitation in non-equilibrium systems is beyond the scope of this review, but can be found in [42,43]. Here, we exclusively use the notion entropy maximization as the principle of minimal information content in the probability model consistent with the data.

### Categorical random variables

In the following section, we derive the pairwise maximum-entropy probability distribution on categorical variables. For jointly distributed categorical variables **x** = (*x*_1_,…, *x*_*L*_)^T^ ∈Ω^*L*^, each variable *x*_*i*_ is defined on the finite set Ω = {*σ*_1_,…, *σ*_*q*_} consisting of *q* elements. In the concrete example of modeling protein co-evolution, this set contains the 20 amino acids represented by a 20-letter alphabet from *A* standing for Alanine to *Y* for Tyrosine plus one gap element, then Ω = {*A*, *C*, *D*, *E*, *F*, *G*, *H*, *I*, *K*, *L*, *M*, *N*, *P*, *Q*, *R*, *S*, *T*, *V*, *W*, *Y*, −} and *q* = 21. Our goal is to extract co-evolving residue pairs from the evolutionary record of a given protein family. As input data, we use a so-called multiple sequence alignment, {**x** ^1^,…, **x** ^*M*^} ⊂Ω*^L×M^*, a collection of closely homologous protein sequences that is formatted such that it allows comparison of the evolution across each residue [44]. These alignments may stem from different hidden Markov model-derived resources, such as PFAM [45], hhblits [46], and Jackhmmer [47].

**Fig 2:**
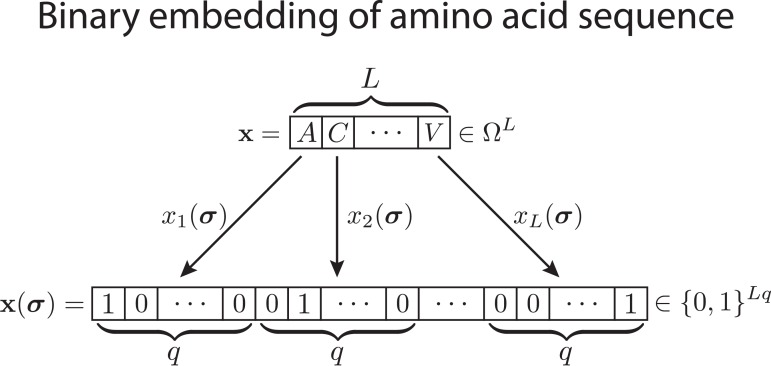
Illustration of binary embedding. The binary embedding **1**_σ_: Ω → {0, 1}^*Lq*^ maps each vector of categorical random variables, **x**∈Ω^*L*^, here represented by a sequence of amino acids from the amino acid alphabet (containing the 20 amino acids and one gap element), Ω = {*A*, *C*, *D*, *E*, *F*, *G*, *H*, *I*, *K*, *L*, *M*, *N*, *P*, *Q*, *R*, *S*, *T*, *V*, *W*, *Y*, −}, onto a unique binary representation, **x(*σ*)**∈{0, 1}^*Lq*^.

To formalize the derivation of the pairwise maximum-entropy probability distribution on categorical variables, we use the approach of [8,30,48] and replace, as depicted in Fig 2, each variable *x*_*i*_ defined on categorical variables by an indicator function of the amino acid *σ* ∈ Ω, **1**_*σ*_: Ω → {0, 1}^*q*^,

$$x_{i}\mapsto x_{i}(\sigma ):\equiv 1_{\sigma }(x_{i})=\{\begin{array}{ll} 1 & \text{if }x_{i}=\sigma ,\\ 0 & \text{otherwise}. \end{array}$$

This embedding specifies a unique representation of any *L*-vector of categorical random variables, **x**, as a binary *Lq*-vector, **x(*σ*)** with a single non-zero entry in each binary *q*-subvector *x*_*i*_ **(*σ*)** = (*x*_*i*_(*σ*_1_),…, *x*_*i*_(*σ*_*q*_))^T^ ∈{0,1}^*q*^,

$$x={\left(x_{1},\ldots ,x_{L}\right)}^{\text{T}}\in {\text{Ω}}^{L}\overset{1_{\sigma }}{\mapsto }x(\sigma )={\left(x_{1}(\sigma_{1}),\ldots ,x_{L}(\sigma_{q})\right)}^{\text{T}}\in {\left\{0,1\right\}}^{Lq}.$$

Inserting this embedding into the first and second moment constraints, corresponding to Eqs 3 and 4 in the continuous variable case, we find their embedded analogues, the single and pairwise marginal probability in positions *i* and *j* for amino acids *σ*,*ω*,∈Ω

$$\langle x_{i}(\sigma )\rangle =\sum_{x(\sigma )}P(x(\sigma ))x_{i}(\sigma )=\sum_{x}P(x_{i}=\sigma )=P_{i}(\sigma ),$$

$$\langle x_{i}(\sigma )x_{j}(\omega )\rangle =\sum_{x(\sigma )}P(x(\sigma ))x_{i}(\sigma )x_{j}(\omega )=\sum_{x}P(x_{i}=\sigma ,x_{j}=\omega )=P_{ij}(\sigma ,\omega )$$

including *P_ii_*(*σ*,*ω*) = *P_i_*(*σ*)**1**_*σ*_(*ω*) and with the distribution’s first moment in each random variable, $\langle y_{i}\rangle =\sum_{y}P(y)y_{i}$ and ***y*** = (*y*_1_,…, *y_Lq_*)^T^ ∈ℝ^*Lq*^. The analogue of the covariance matrix then becomes a symmetric *Lq* × *Lq* matrix of connected correlations whose entries *C*_*ij*_(*σ*,*ω*) = *P*_*ij*_(*σ*,*ω*) − *P*_*i*_(*σ*) *P*_*j*_(*ω*) characterize the dependencies between pairs of variables. In the same way, the sample means translate to the single-site and pair frequency counts over *m* = 1,…, *M* data vectors $x^{m}={\left(x_{1}^{m},\ldots ,x_{L}^{m}\right)}^{\text{T}}\in \Omega^{L}$,

$$\bar{x_{i}(\sigma )}=\frac{1}{M}\sum_{m=1}^{M}x_{i}^{m}(\sigma )=f_{i}(\sigma ),$$

$$\bar{x_{i}(\sigma )x_{j}(\omega )}=\frac{1}{M}\sum_{m=1}^{M}x_{i}^{m}(\sigma )x_{j}^{m}(\omega )=f_{ij}(\sigma ,\omega ).$$

The pairwise maximum-entropy probability distribution in categorical variables has to fulfill the normalization constraint,

$$\sum_{x}P(x)=\sum_{x(\sigma )}P(x(\sigma ))=1.$$ (9)

Furthermore, the single and pair constraints, the analogues of Eqs 3 and 4, enforce the resulting probability distribution to be compatible with the measured single and pair frequency counts,

$$P_{i}(\sigma )=f_{i}(\sigma ),\;P_{ij}(\sigma ,\omega )=f_{ij}(\sigma ,\omega )$$ (10)

for each *i*, *j* = 1,…, *L* and amino acids *σ*,*ω*∈Ω. As before, we require the probability distribution to maximize the information entropy,

$$\text{maximize }S=-\sum_{x}P(x)\text{ln }P(x)=-\sum_{x(\sigma )}P\left(x(\sigma )\right)\text{ln }P(x(\sigma )).$$ (11)

The corresponding Lagrangian, ℒ=ℒ(P(x(σ));α,β(σ),γ(σ,ω)), has the functional form,

$$ℒ=S+\alpha (\langle 1\rangle -1)+\sum_{i=1}^{L}\sum_{\sigma \in \Omega }\beta_{i}(\sigma )\left(P_{i}(\sigma )-f_{i}(\sigma )\right)+\sum_{i,j=1}^{L}\sum_{\sigma ,\omega \in \Omega }\gamma_{ij}(\sigma ,\omega )\left(P_{ij}(\sigma ,\omega )-f_{ij}(\sigma ,\omega )\right).$$

For notational convenience, the Lagrange multipliers *β*_*i*_(*σ*) and *γ*_*ij*_(*σ*,*ω*) are grouped to the *Lq*-vector $\beta (\sigma )=(\beta_{i}(\sigma {))}_{i=1,\ldots ,L}^{\sigma \in \Omega }$ and the *Lq* × *Lq*-matrix $\gamma (\sigma ,\omega )=(\gamma_{ij}(\sigma ,\omega {))}_{i,j=1,\ldots ,L}^{\sigma ,\omega \in \Omega }$, respectively. The Lagrangian’s stationary point, found as the solution of $\frac{\partial ℒ}{\partial P(x(\sigma ))}=0$, determines the pairwise maximum-entropy probability distribution in categorical variables [30,49],

$$P(x(\sigma );\beta ,\gamma )=\frac{1}{Z}\text{exp}\left(\sum_{i=1}^{L}\sum_{\sigma \in \Omega }\beta_{i}(\sigma )x_{i}(\sigma )+\sum_{i,j=1}^{L}\sum_{\sigma ,\omega \in \Omega }\gamma_{ij}(\sigma ,\omega )x_{i}(\sigma )x_{j}(\omega )\right)$$ (12)

with normalization by the partition function, *Z* ≡ exp(1−*α*). Note that distribution Eq 12 is of the same functional form as Eq 8 but with binary random variables **x(*σ*)** ∈{0,1}^*Lq*^ instead of continuous ones **x**∈ℝ^*L*^. At this point, we introduce the reduced parameter set, *h*_*i*_(*σ*): = *β*_*i*_(*σ*)+*γ*_*ii*_(*σ*, *σ*) and *e*_*ij*_(*σ*,*ω*): = 2*γ*_*ij*_(*σ*,ω) for *i* < *j*, using the symmetry of the Lagrange multipliers, *γ*_*ij*_(*σ*,*ω*): = *γ*_*ji*_(ω, *σ*), and that *x*_*i*_(*σ*) *x*_*i*_(*ω*) = 1 if and only if *σ* = *ω*. For a given sequence (*z*_1_,…, *z*_*L*_)∈Ω^*L*^ summing over all non-zero elements, (*x*_1_(*z*_1_) = 1,…, *x*_*L*_(*z*_*L*_) = 1) or equivalently (*x*_1_ = *z*_1_,…, *x*_*L*_ = *z*_*L*_) then yields the probability assigned to the sequence of interest,

$$P(z_{1},\ldots ,z_{L})\equiv \frac{1}{Z}\text{exp}\left(\sum_{i=1}^{L}h_{i}(z_{i})+\sum_{1\leq i<j\leq L}e_{ij}(z_{i},z_{j})\right).$$ (13)

This is the 21-state maximum-entropy probability distribution as presented by [5–7].

### Gauge fixing

In contrast to the continuous variable case in which the number of constraints naturally matches the number of unknown parameters, the case of categorical variables has dependencies due to $1=\sum_{\sigma \in \Omega }P_{i}(\sigma )$ for each *i* = 1,…, *L* and $P_{i}(\sigma )=\sum_{\omega \in \Omega }P_{ij}(\sigma ,\omega )$ for each *i*, *j* = 1,…, *L* and *σ*∈Ω. This results in at most $\frac{L(L-1)}{2}{\left(q-1\right)}^{2}+L(q-1)$ independent constraints compared to $\frac{L(L-1)}{2}q^{2}+Lq$ free parameters to be estimated. To ensure the uniqueness of the inferred parameters in defining the Hamiltonian, $ℋ(x_{1},\ldots ,x_{L})=-\sum_{i<j}e_{ij}(x_{i},x_{j})-\sum_{i}h_{i}(x_{i})$, and, by implication, the probability distribution, one has to reduce the number of independent parameters such that these match the number of independent constraints. For this purpose, so-called gauge fixing [5] has been proposed, which can be realized in different ways. For example, the authors of [6,7] set the parameters corresponding to the last amino acid in the alphabet, *σ*_*q*_, to zero, i.e., *e*_*ij*_(*σ*_*q*_, ·) = *e*_*ij*_(·, *σ*_*q*_) = 0 and *h*_*i*_(*σ*_*q*_) = 0 for 1 ≤ *i* < *j* ≤ *L*, resulting in rows and columns of zeros at the end of each q × q -block of the *Lq × Lq* coupling matrix. Alternatively, the authors of [5] introduce a zero-sum gauge, $\underset{\sigma }{\sum }e_{ij}(\sigma ,\omega )=\underset{\sigma }{\sum }e_{ij}(\omega^{\prime },\sigma )=0$ and $\sum_{\sigma }h_{i}(\sigma )=0$ for each 1 ≤ *i* < *j* ≤ *L* and *ω* ^′^∈Ω. However, different gauge fixings are not equally efficient for the purpose of protein contact prediction. The zero-sum gauge is the parameter fixing that minimizes the sum of squares of the pairwise parameters in the Hamiltonian ℋ, $\sum_{\sigma ,\omega }e_{ij}{\left(\sigma ,\omega \right)}^{2}$, which makes it the suitable choice when using non-gauge invariant scoring functions, such as the (average product-corrected) Frobenius norm [5,50] (see section “Scoring Functions”). Moreover, no gauge fixing is required when combining the strictly convex *ℓ* ^1^- or *ℓ* ^2^-regularizer with negative loglikelihood minimization; here the regularizer selects for a unique representation among all parametrizations of the optimal distribution [32,51]. However, to additionally minimize the Frobenius norm of the pairwise interactions, [51] changed the obtained full parameter set from regularized inference with plmDCA to zero-sum gauge by, $e_{ij}(\sigma ,\omega )\mapsto e_{ij}(\sigma ,\omega )-\frac{1}{q}\sum_{\sigma^{\prime }}e_{ij}(\sigma^{\prime },\omega )-\frac{1}{q}\sum_{\omega^{\prime }}e_{ij}(\sigma ,\omega^{\prime })+\frac{1}{q^{2}}\sum_{\sigma^{\prime },\omega^{\prime }}e_{ij}(\sigma^{\prime },\omega^{\prime })$, where *q* denotes the length of the alphabet.

### Network interpretation

The derived pairwise maximum-entropy distributions in Eqs 13 or 12 and 8 specify an undirected graphical model or Markov random field [34,41]. In particular, a graphical model represents a probability distribution in terms of a graph that consists of a node and an edge set. Edges characterize the dependence structure between nodes and a missing edge then corresponds to **conditional independence** given the remaining random variables. For continuous, real-valued variables, the maximum-entropy distribution with first and second moment constraints is multivariate Gaussian, which will be demonstrated in the next section. Its dependency structure is represented by a graphical Gaussian model (GGM) in which a missing edge, *γ*_*ij*_ = 0, corresponds to conditional independence between the random variables *x*_*i*_ and *x*_*j*_ (given the remaining ones), and is further specified by a zero entry in the corresponding inverse covariance matrix, (*C* ^−1^)_*ij*_ = 0.

In the next section, we describe how the dependency structure of the graph is inferred.

## Inference of Interactions

Up to this point, the functional form of the maximum-entropy probability distribution is specified, but not its determining parameters. For categorical variables with dimension *L* > 1, there is typically no closed-form solution. In the following section, we present several inference methods to estimate these parameters that have recently been used in the context of protein contact prediction. Those are (1) for continuous variables, the exact closed-form solution which approximates the mean-field result for categorical variables, and (2) three inference methods for categorical variables based on the maximum-likelihood methodology: the stochastic maximum likelihood, the approximation by pseudo-likelihood maximization, and finally, the sparse maximum-likelihood solution.

## Closed-Form Solution for Continuous Variables

The simplest approach to extract the unknown Lagrange multipliers *α*, ***β*** = (*β*_*i*_), and ***γ*** = (*γ*_*ij*_) from *P*(**x**) exactly is to use basic integration properties of the continuous random variables *x*_*i*_ in the constraints Eqs 3–5. For this purpose, we rewrite the exponent of the pairwise maximum-entropy probability distribution Eq 8,

$$P(x;\beta ,\tilde{\gamma })=\frac{1}{Z}\text{exp}\left(\beta^{\text{T}}x-\frac{1}{2}x^{\text{T}}\tilde{\gamma }x\right)=\frac{1}{Z}\text{exp}\left(\frac{1}{2}\beta^{\text{T}}{\tilde{\gamma }}^{-1}\beta -\frac{1}{2}{\left(x-{\tilde{\gamma }}^{-1}\beta \right)}^{\text{T}}\tilde{\gamma }\left(x-{\tilde{\gamma }}^{-1}\beta \right)\right),$$

where we use the replacement $\tilde{\gamma }:=-2\gamma$ and require $\tilde{\gamma }$ to be positive definite (which is equivalent to ***γ*** being negative definite), i.e., $x^{\text{T}}\tilde{\gamma }x>0$ for any **x** ≠ **0**, which makes its inverse ${\tilde{\gamma }}^{-1}=-\frac{1}{2}\gamma^{-1}$ well-defined. As already discussed, this is a sufficient condition on the integrals in Eqs 3–6 to be finite. For notational convenience, we define the shifted variable $z={\left(z_{1},\ldots ,z_{L}\right)}^{\text{T}}:=x-{\tilde{\gamma }}^{-1}\beta$ or $x_{i}=z_{i}+\sum_{j=1}^{L}{\left({\tilde{\gamma }}^{-1}\right)}_{ij}\beta_{j}$ and accordingly, the maximum-entropy probability distribution becomes

$$P(x)=\frac{1}{\tilde{Z}}\text{exp}\left(-\frac{1}{2}{\left(x-{\tilde{\gamma }}^{-1}\beta \right)}^{\text{T}}\tilde{\gamma }\left(x-{\tilde{\gamma }}^{-1}\beta \right)\right)\equiv \frac{1}{\tilde{Z}}{\text{e}}^{-\frac{1}{2}z^{\text{T}}\tilde{\gamma }z}$$ (14)

with the normalization constant $\tilde{Z}=\text{exp}\left(1-\alpha -\frac{1}{2}\beta^{\text{T}}{\tilde{\gamma }}^{-1}\beta \right)$. The normalization condition Eq 3 in the new variable is,

$$1=\int_{x}P(x)\text{ d}x\equiv \frac{1}{\tilde{Z}}\int_{z}{\text{e}}^{-\frac{1}{2}z^{\text{T}}\tilde{\gamma }z}\text{ d}z$$ (15)

and the linear shift does not affect the integral when integrated over ℝ^*L*^ yielding for the normalization constant,$\tilde{Z}=\int_{z}{\text{e}}^{-\frac{1}{2}z^{\text{T}}\tilde{\gamma }z}\text{ d}z$. Furthermore, the first-order constraint Eq 4 becomes for each *i* = 1,…, *L*,

$$\langle x_{i}\rangle =\int_{x}P(x)x_{i}\text{ d}x\equiv \frac{1}{\tilde{Z}}\int_{z}{\text{e}}^{-\frac{1}{2}z^{\text{T}}\tilde{\gamma }z}\left(z_{i}+\sum_{j=1}^{L}{\left({\tilde{\gamma }}^{-1}\right)}_{ij}\beta_{j}\right)\text{ d}z=\sum_{j=1}^{L}{\left({\tilde{\gamma }}^{-1}\right)}_{ij}\beta_{j}$$

and we used the point symmetry of the integrand then, $\int_{z}{\text{e}}^{-\frac{1}{2}z^{\text{T}}\tilde{\gamma }z}z_{i}\text{ d}z=0$ in each *i* = 1,…, *L*. Analogously, we find for the second moment, determining the correlations for each index pair *i*, *j* = 1,…, *L*,

$$\langle x_{i}x_{j}\rangle =\int_{x}P(x)x_{i}x_{j}\text{ d}x\equiv \frac{1}{\tilde{Z}}\int_{z}{\text{e}}^{-\frac{1}{2}z^{\text{T}}\tilde{\gamma }z}\left(z_{i}-\langle x_{i}\rangle \right)\left(z_{j}-\langle x_{j}\rangle \right)\text{ d}z=\langle z_{i}z_{j}\rangle +\langle x_{i}\rangle \langle x_{j}\rangle ,$$

where we use again the point symmetry and the result on the normalization constraint. Based on this, the covariance is found as,

$$C_{ij}=\langle x_{i}x_{j}\rangle -\langle x_{i}\rangle \langle x_{j}\rangle \equiv \langle z_{i}z_{j}\rangle .$$

Finally, the term 〈*z*_*i*_ *z*_*j*_〉 is solved using a spectral decomposition of the symmetric and positive-definite matrix $\tilde{\gamma }$ as sum over products of its eigenvectors **v**_1_,…, **v**_*L*_ and real-valued and positive eigenvalues *λ*_1_,…, *λ*_*L*_,$\tilde{\gamma }=\sum_{k=1}^{L}\lambda_{k}v_{k}v_{k}^{\text{T}}$. The eigenvectors form a basis of ℝ^*L*^ and assign new coordinates, *y*_1_,…, *y*_*L*_, to $z=\sum_{k=1}^{L}y_{k}v_{k}$, which allows writing of the exponent 〈*z*_*i*_ *z*_*j*_〉 as $z^{\text{T}}\tilde{\gamma }z=\sum_{k=1}^{L}\lambda_{k}y_{k}^{2}$. The covariance between *x*_*i*_ and *x*_*j*_ then reads as (Bishop [52], p. 83)

$$\langle z_{i}z_{j}\rangle =\frac{1}{\tilde{Z}}\sum_{l,n=1}^{L}{\left(v_{l}\right)}_{i}{\left(v_{n}\right)}_{j}\int_{y}\text{exp}\left(-\frac{1}{2}\sum_{k=1}^{L}\lambda_{k}y_{k}^{2}\right)y_{l}y_{n}\text{ d}y=\sum_{k=1}^{L}\frac{1}{\lambda_{k}}{\left(v_{k}\right)}_{i}{\left(v_{k}\right)}_{j}\equiv {\left({\tilde{\gamma }}^{-1}\right)}_{ij}$$

with solution $C_{ij}={\left({\tilde{\gamma }}^{-1}\right)}_{ij}$ or ${\left(C^{-1}\right)}_{ij}={\left(\tilde{\gamma }\right)}_{ij}=-2\gamma_{ij}$. Taken together, the Lagrange multipliers ***β*** and ***γ*** are specified in terms of the mean, 〈**x**〉, and the inverse covariance matrix (also known as the precision or concentration matrix), *C* ^−1^,

$$\beta =C^{-1}\langle x\rangle ,\;\gamma =-\frac{1}{2}\tilde{\gamma }=-\frac{1}{2}C^{-1}.$$ (16)

As a consequence, the real-valued maximum-entropy distribution Eq 14 for given first and second moments is found as the **multivariate Gaussian distribution,** which is determined by the mean 〈**x**〉 and the covariance matrix *C*,

$$P(x;\langle x\rangle ,C)={\left(2\pi \right)}^{-L/2}\text{det}{\left(C\right)}^{-1/2}\text{exp}\left(-\frac{1}{2}{\left(x-\langle x\rangle \right)}^{\text{T}}C^{-1}\left(x-\langle x\rangle \right)\right)$$ (17)

and we refer to [52] for the derivation of the normalization factor. The initial requirement of $\tilde{\gamma }=-2\gamma$ to be positive definite results in a positive-definite covariance matrix *C*, a necessary condition for the Gaussian density to be well defined. In summary, the multivariate Gaussian distribution maximizes the entropy among all probability distributions of continuous variables with specified first and second moments. The pair interaction strength is now evaluated by the already introduced partial correlation coefficient between *x*_*i*_ and *x*_*j*_ given the remaining variables {*x*_*r*_}_*r*∈{1,…, *L*}\{*i,j*}_,

$$\rho_{ij\cdot \{1,\ldots ,L\}\backslash \{i,j\}}\equiv \frac{\gamma_{ij}}{\sqrt{\gamma_{ii}\gamma_{jj}}}=\{\begin{array}{cc} -\frac{{\left(C^{-1}\right)}_{ij}}{\sqrt{{\left(C^{-1}\right)}_{ii}{\left(C^{-1}\right)}_{jj}}} & \text{if }i\neq j,\\ 1 & \text{if }i=j. \end{array}$$ (18)

### Data integration

In biological datasets as used to study gene association, the number of measurements, *M*, is typically smaller than the number of observables, *L*, i.e., *M* < *L* in our terminology. Consequently, the empirical covariance matrix, $\hat{C}=\frac{1}{M}\sum_{m=1}^{M}\left(x^{m}-\bar{x}\right){\left(x^{m}-\bar{x}\right)}^{\text{T}}$, will in these cases always be rank-deficient (and, thus, not invertible) since its rank can exceed neither the number of variables, *L*, nor the number of measurements, *M*. Moreover, even in cases when *M* ≥ *L*, the empirical covariance matrix may become non-invertible or badly conditioned (i.e., close to singular) due to dependencies in the data. However, for variables following a multivariate Gaussian distribution, one can access the elements of its inverse by maximizing the penalized Gaussian loglikelihood, which results in the following estimate of the inverse covariance matrix, $C^{-1}\approx C_{\delta ,\lambda }^{-1}$,

$$C_{\delta ,\lambda }^{-1}=\underset{\begin{array}{c} \Theta \text{ pos. definite},\\ \text{symmetric} \end{array}}{\text{arg max}}\left\{\text{ln det}(\Theta )-\text{trace}(\hat{C}\Theta )-\lambda ∥\Theta {∥}_{\delta }^{\delta }\right\}$$ (19)

with penalty parameter *λ* ≥ 0 and $∥\Theta {∥}_{\delta }^{\delta }=\sum_{i,j}|\Theta_{ij}{|}^{\delta }$. If *λ* = 0, we obtain the maximum-likelihood estimate, for *δ* = 1 and *λ* > 0 the *ℓ* ^1^-regularized (sparse) maximum-likelihood solution that selects for sparsity [53,54], and for *δ* = 2 and *λ* > 0 the *ℓ* ^2^-regularized maximum-likelihood solution that favors small absolute values in the entries of the selected inverse covariance matrix [55]. For *δ* = 1 and *λ* > 0, the method is called LASSO, for *δ* = 2 and *λ* > 0, ridge regression. Alternatively, regularization can be directly applied to the covariance matrix, e.g., by shrinkage [17,56].

### Solution for categorical variables

An ad hoc ansatz to extract the pairwise parameters in the categorical variables case (12) is to extend the binary variable $x(\sigma )=(x_{i}(\sigma_{k}{))}_{i\cdot k}\in {\left\{0,1\right\}}^{L(q-1)}$ to a continuous one, **y** = (*y*_*j*_)_*j*_ ∈ℝ^L(q−1)^, and replace the sums in the distribution and the moments 〈·〉 by integrals. The extended binary maximum-entropy distribution Eq 12 is then approximated by the *Lq*-dimensional multivariate Gaussian with inherited analogues of the mean $\langle y\rangle ={\left(f_{i}(\sigma_{k})\right)}_{i\cdot k}\in {\mathbb{R}}^{L(q-1)}$ and the empirical covariance matrix $\hat{C}(\sigma ,\omega )=({\hat{C}}_{ij}(\sigma_{k},\sigma_{l}{))}_{i,j,k,l}\in {\mathbb{R}}^{L(q-1)\times L(q-1)}$ whose elements ${\hat{C}}_{ij}(\sigma ,\omega )=f_{ij}(\sigma ,\omega )-f_{i}(\sigma )f_{j}(\omega )$ are characterizing the pairwise dependency structure. The gauge fixing results in setting the preassigned entries referring to the last amino acid in the mean vector and the covariance matrix to zero, which reduces the model’s dimension from *Lq* to *L*(*q*−1); otherwise the unregularized covariance matrix would always be non-invertible. Typically, the single and pair frequency counts are reweighted and regularized by pseudocounts (see section “Sequence data preprocessing”) to additionally ensure that $\hat{C}(\sigma ,\omega )$ is invertible. Final application of the closed-form solution for continuous variables Eq 16 to the extended binary variables for $C^{-1}(\sigma ,\omega )\approx {\hat{C}}^{-1}(\sigma ,\omega )$ yields the so-called mean-field (MF) approximation [48],

$$\gamma_{ij}^{\text{MF}}(\sigma ,\omega )=-\frac{1}{2}{\left(C^{-1}\right)}_{ij}(\sigma ,\omega )\;⟹\;e_{ij}^{\text{MF}}(\sigma ,\omega )=-{\left(C^{-1}\right)}_{ij}(\sigma ,\omega )$$ (20)

for amino acids *σ*,*ω*∈Ω and with restriction to residues *i* < *j* in the latter identity. The same solution has been obtained by [6,7] using a perturbation ansatz to solve the *q*-state Potts model termed (mean-field) Direct Coupling Analysis (DCA or mfDCA). In Ising models, this result is also known as naïve mean-field approximation [57–59].

The following section is dedicated to maximum likelihood-based inference approaches, which have been presented in the field of protein contact prediction.

## Maximum-Likelihood Inference

A well-known approach to estimate the parameters of a model is maximum-likelihood inference. The likelihood is a scalar measure of how likely the model parameters are, given the observed data (Mackay [34], p. 29), and the maximum-likelihood solution denotes the parameter set maximizing the likelihood function. For Markov random fields, the maximum-likelihood solution is consistent, i.e., recovers the true model parameters in the limit of infinite data (Koller and Friedman [32], p. 949). In particular, for a pairwise model with parameters $h(\sigma )=(h_{i}(\sigma {))}_{i=1,\ldots ,L}^{\sigma \in \Omega }$ and $e(\sigma ,\omega )=(e_{ij}(\sigma ,\omega {))}_{1\leq i<j\leq L}^{\sigma ,\omega \in \Omega }$, we find the likelihood *l*(***h***(***σ***),***e***(***σ*,*ω***)) = *l*(***h***(***σ***),***e***(***σ*,*ω***)|**x** ^1^,…, **x** ^*M*^) given observed data, **x** ^1^,…, **x** ^*M*^ ∈Ω^*L*^, which are assumed to be independent and identically distributed (iid), as

$$l\left(h(\sigma ),e(\sigma ,\omega )|x^{1},\ldots ,x^{M}\right)=\prod_{m=1}^{M}P(x^{m};h(\sigma ),e(\sigma ,\omega )).$$ (21)

The estimates of the model parameters are then obtained as the maximizer of *l* or, using the monotonicity of the logarithm, the minimizer of —ln l,

$$\{h^{\text{ML}}(\sigma ),e^{\text{ML}}(\sigma ,\omega )\}=\underset{h(\sigma ),e(\sigma ,\omega )}{\text{arg max}}\;l(h(\sigma ),e(\sigma ,\omega ))\equiv \underset{h(\sigma ),e(\sigma ,\omega )}{\text{arg min}}\;-\text{ln }l(h(\sigma ),e(\sigma ,\omega )).$$

When we specify the maximum-entropy distribution Eq 13 as model distribution, the then-concave loglikelihood [32] becomes

$$\begin{array}{l} \text{ln }l(h(\sigma ),e(\sigma ,\omega ))=\sum_{m=1}^{M}\text{ln }P(x^{m};h(\sigma ),e(\sigma ,\omega ))\\ =-M\left[\text{ln }Z-\sum_{i=1}^{L}\sum_{\sigma }h_{i}(\sigma )f_{i}(\sigma )-\sum_{1\leq i<j\leq L}\sum_{\sigma ,\omega }e_{ij}(\sigma ,\omega )f_{ij}(\sigma ,\omega )\right]. \end{array}$$ (22)

The maximum-likelihood solution is found by taking the derivatives of Eq 22 with respect to the model parameters *h*_*i*_(*σ*) and *e*_*ij*_(*σ*,*ω*) and setting to zero,

$$\begin{array}{ll} \frac{\partial }{\partial h_{i}(\sigma )}\text{ln }l & =-M\left[{\frac{\partial }{\partial h_{i}(\sigma )}\text{ln }Z|}_{\left\{h(\sigma ),e(\sigma ,\omega )\right\}}-f_{i}(\sigma )\right]=0,\\ \frac{\partial }{\partial e_{ij}(\sigma ,\omega )}\text{ln }l & =-M\left[{\frac{\partial }{\partial e_{ij}(\sigma ,\omega )}\text{ln }Z|}_{\left\{h(\sigma ),e(\sigma ,\omega )\right\}}-f_{ij}\left(\sigma ,\omega \right)\right]=0. \end{array}$$ (23)

The partial derivatives of the partition function, $Z=\sum_{\left(x_{1},\ldots ,x_{L}\right)}\text{exp}\left(\sum_{i}h_{i}(x_{i})+\sum_{i<j}e_{ij}(x_{i},x_{j})\right)$, follow the well-known identities

$${\frac{\partial }{\partial h_{i}(\sigma )}\text{ln }Z|}_{\left\{h(\sigma ),e(\sigma ,\omega )\right\}}=\frac{1}{Z}\partial_{h_{i}(\sigma )}{Z|}_{\left\{h(\sigma ),e(\sigma ,\omega )\right\}}=P_{i}\left(\sigma ;h(\sigma ),e(\sigma ,\omega )\right),$$

$${\frac{\partial }{\partial e_{ij}(\sigma ,\omega )}\text{ln }Z|}_{\left\{h(\sigma ),e(\sigma ,\omega )\right\}}=\frac{1}{Z}\partial_{e_{ij}(\sigma ,\omega )}{Z|}_{\left\{h(\sigma ),e(\sigma ,\omega )\right\}}=P_{ij}\left(\sigma ,\omega ;h(\sigma ),e(\sigma ,\omega )\right).$$

The maximizing parameters, $h^{\text{ML}}(\sigma )={\left(h_{i}^{\text{ML}}(\sigma )\right)}_{i=1,\ldots ,L}^{\sigma \in \Omega }$ and $e^{\text{ML}}(\sigma ,\omega )={\left(e_{ij}^{\text{ML}}(\sigma ,\omega )\right)}_{1\leq i<j\leq L}^{\sigma ,\omega \in \Omega }$, are those matching the distribution’s single and pair marginal probabilities with the empirical single and pair frequency counts,

$$P_{i}(\sigma ;h^{\text{ML}}(\sigma ),e^{\text{ML}}(\sigma ,\omega ))=f_{i}(\sigma ),\;P_{ij}(\sigma ,\omega ;h^{\text{ML}}(\sigma ),e^{\text{ML}}(\sigma ,\omega ))=f_{ij}(\sigma ,\omega )$$

in residues *i* = 1,…, *L* and *i*,*j* = 1,…, *L*, respectively, and for amino acids *σ*,*ω*∈Ω. In other words, matching the moments of the pairwise maximum-entropy probability distribution to the given data is equivalent to maximum-likelihood fitting of an exponential family [34,60]. Although the maximum-likelihood solution is globally optimal for the pairwise maximum-entropy probability model, based on the concavity of ln *l*, the resulting distribution is not necessarily unique, due to dependencies in the input data (Koller and Friedman [32], p. 948). To remove these equivalent optima and select for a unique representation, one needs to introduce further constraints by, for example, gauge fixing or regularization.

Based on the maximum-likelihood principle, we present three solution approaches in the remainder of this section.

### Stochastic maximum likelihood

The maximum-likelihood solution is typically inaccessible for models of categorical variables due to the computational complexity of estimating the partition function *Z* which involves a sum over all possible states and grows exponentially with the size of the system [3,61]. Lapedes et al. [30] solved Eq 22 by likelihood maximization on sampled subsets using the Metropolis–Hastings algorithm [32,34]. In particular, the likelihood is maximized iteratively by following the steepest ascent of the loglikelihood function ln *l* using Eq 23. In each maximization step, the parameters $h_{i}^{\left(k\right)}(\sigma )$ and $e_{ij}^{\left(k\right)}(\sigma ,\omega )$ are changed in proportion to the gradient of ln *l* and scaled by the constant step size *ε* > 0,

$$\Delta h_{i}^{\left(k\right)}(\sigma )=\varepsilon {\frac{\partial }{\partial h_{i}(\sigma )}\text{ln }l|}_{\left\{h^{\left(k\right)}(\sigma ),e^{\left(k\right)}(\sigma ,\omega )\right\}}\propto f_{i}(\sigma )-P_{i}(\sigma ;h^{\left(k\right)}(\sigma ),e^{\left(k\right)}(\sigma ,\omega )),$$

$$\Delta e_{ij}^{\left(k\right)}(\sigma ,\omega )=\varepsilon {\frac{\partial }{\partial e_{ij}(\sigma ,\omega )}\text{ln }l|}_{\left\{h^{\left(k\right)}(\sigma ),e^{\left(k\right)}(\sigma ,\omega )\right\}}\propto f_{ij}(\sigma ,\omega )-P_{ij}(\sigma ,\omega ;h^{\left(k\right)}(\sigma ),e^{\left(k\right)}(\sigma ,\omega ))$$

until convergence is reached as the differences $\Delta h_{i}^{\left(k\right)}(\sigma ,\omega ):=h_{i}^{\left(k+1\right)}(\sigma ,\omega )-h_{i}^{\left(k\right)}(\sigma ,\omega )$, *i* = 1,…, *L*, and $\Delta e_{ij}^{\left(k\right)}(\sigma ,\omega ):=e_{ij}^{\left(k+1\right)}(\sigma ,\omega )-e_{ij}^{\left(k\right)}(\sigma ,\omega )$, 1 ≤ *i* < *j* ≤ *L*, go to zero [30]. The computation of the marginals requires summing over 20^*L*^ states and is, for example, estimated by Monte-Carlo sampling. As the likelihood is concave, there are no local maxima and the maximum-likelihood parameters are obtained in the limit k→∞,

$$\left\{h^{\text{ML}}(\sigma ),e^{\text{ML}}(\sigma ,\omega )\}=\underset{k\to \infty }{\text{lim}}\;\{h^{\left(k\right)}(\sigma ),e^{\left(k\right)}(\sigma ,\omega )\right\}$$

or $\Delta h_{i}^{\left(k\right)}(\sigma ,\omega )\to 0$ for *i* = 1,…, *L* and $\Delta e_{ij}^{\left(k\right)}(\sigma ,\omega )\to 0$ for 1 ≤ *i* < *j* ≤ *L* and *σ*,*ω*∈Ω \ {*σ*_*q*_}, a subset of Ω containing *q*−1 elements to account for gauge fixing.

### Pseudo-likelihood maximization

Besag [62] introduced the pseudo-likelihood as approximation to the likelihood function in which the global partition function is replaced by computationally tractable local estimates. The pseudo-likelihood inherits the concavity from the likelihood and yields the exact maximum-likelihood parameter in the limit of infinite data for Gaussian Markov random fields [41,62], but not in general [63]. Applications of this approximation to non-continuous categorical variables have been studied, for instance, in sparse inference of Ising models [64] but may lead to results that differ from the maximum-likelihood estimate. In this approach, the probability of the *m*-th observation, **x** ^*m*^, is approximated by the product of the conditional probabilities of $x_{r}=x_{r}^{m}$ given observations in the remaining variables $x_{\backslash r}:={\left(x_{1},\ldots ,x_{r-1},x_{r+1},\ldots ,x_{L}\right)}^{\text{T}}\in \Omega^{L-1}$ [51],

$$P\left(x^{m};h(\sigma ),e(\sigma ,\omega )\right)≃\prod_{r=1}^{L}P(x_{r}=x_{r}^{m}|x_{\backslash r}=x_{\backslash r}^{m};h(\sigma ),e(\sigma ,\omega )).$$

Each factor is of the following analytical form,

$$P(x_{r}=x_{r}^{m}|x_{\backslash r}=x_{\backslash r}^{m};h(\sigma ),e(\sigma ,\omega ))=\frac{\text{exp}\left(h_{r}(x_{r}^{m})+\sum_{j\neq r}e_{rj}(x_{r}^{m},x_{j}^{m})\right)}{\sum_{\sigma }\text{exp}\left(h_{r}\left(\sigma \right)+\sum_{j\neq r}e_{rj}(\sigma ,x_{j}^{m})\right)},$$

which only depends on the unknown parameters (*e*_*ij*_(*σ*,*ω*))_*i*≠*r*,*j*≠*r*_ and (*h*_*i*_(*σ*))_*i*≠*r*_ and makes the computation of the pseudo-likelihood tractable. Note, we treat *e*_*ij*_(*σ*,*ω*) = *e*_*ji*_(*ω*,*σ*) and *e*_*ii*_(·,·) = 0. By this approximation, the loglikelihood Eq 21 becomes the pseudo-loglikelihood,

$$\text{ln}l_{\text{PL}}(h(\sigma ),e(\sigma ,\omega )):=\sum_{m=1}^{M}\sum_{r=1}^{L}\text{ln }P(x_{r}=x_{r}^{m}|x_{\backslash r}=x_{\backslash r}^{m};h(\sigma ),e(\sigma ,\omega )).$$

In the final formulation of the pseudo-likelihood maximization (PLM) problem, an *ℓ* ^2^-regularizer is added to select for small absolute values of the inferred parameters,

$$\left\{h^{\text{PLM}}(\sigma ),e^{\text{PLM}}(\sigma ,\omega )\right\}=\underset{h(\sigma ),e(\sigma ,\omega )}{\text{arg min}}\left\{-\text{ln}l_{\text{PL}}(h(\sigma ),e(\sigma ,\omega ))+\lambda_{h}∥h(\sigma ){∥}_{2}^{2}+\lambda_{e}∥e(\sigma ,\omega ){∥}_{2}^{2}\right\},$$

where *λ*_***h***_, *λ*_***e***_ > 0 adjust the complexity of problem and are selected in a consistent manner across different protein families to avoid overfitting. This approach has been presented (with scaling of the pseudo-loglikelihood by $\frac{1}{M_{\text{eff}}}w_{m}$ to include sequence weighting, see section “Sequence data preprocessing”) by [51] under the name plmDCA (PseudoLikelihood Maximization Direct Coupling Analysis) and has shown performance improvements compared to the mean-field approximation Eq 20. Another inference method based on the pseudolikelihood maximization but including prior knowledge in terms of secondary structure and information on pairs likely to be in contact is Gremlin (Generative REgularized ModeLs of proteINs) [65–67].

### Sparse maximum likelihood

Similar to the derivation of the mean-field result (20), Jones et al. [8] approximated Eq 12 by a multivariate Gaussian and accessed the elements of the inverse covariance matrix by a maximum-likelihood inference under sparsity constraint [54,68,69]. The corresponding method has been called Psicov (Protein Sparse Inverse COVariance). The validity of this approach to solve the sparse maximum-likelihood problem in binary systems such as Ising models has been demonstrated by [69], followed by consistency studies [70]. In particular, the Psicov method infers the sparse maximum-likelihood estimate of the inverse covariance matrix Eq 19 for *δ* = 1 using the analogue of the empirical covariance matrix derived from the observed amino acid frequencies,$\hat{C}(\sigma ,\omega )$. Its elements ${\hat{C}}_{ij}(\sigma ,\omega )=f_{ij}(\sigma ,\omega )-f_{i}(\sigma )f_{j}(\omega )$, the empirical connected correlations, are preprocessed by reweighting and regularized by pseudocounts and shrinkage. Regularized loglikelihood maximization Eq 19 selects a unique representation of the model, i.e., no additional gauge fixing is required. Using identity Eq 16 on the elements of the sparse maximum-likelihood (SML) estimate of the inverse covariance, $C_{1,\lambda }^{-1}(\sigma ,\omega )$, yields the estimates for the Lagrange multipliers,

$$\gamma_{ij}^{\text{SML}}(\sigma ,\omega )=-\frac{1}{2}{\left(C_{1,\lambda }^{-1}\right)}_{ij}(\sigma ,\omega )\;⟹\;e_{ij}^{\text{SML}}(\sigma ,\omega )=-{\left(C_{1,\lambda }^{-1}\right)}_{ij}(\sigma ,\omega )$$

for *σ*,*ω*∈Ω; in the second identity, the symmetric Lagrange multipliers *γ*_*ij*_(*σ*,*ω*) defined for indices *i*,*j* = 1,…, *L* have been hypothetically translated to the reduced parameter formulation *e*_*ij*_(*σ*,*ω*) for 1 ≤ *i* < *j* ≤ *L*.

### Sequence data preprocessing

The study of residue–residue co-evolution is based on data from multiple sequence alignments, which represent sampling from the evolutionary record of a protein family. Multiple sequence alignments from currently existing sequence databases do not evenly represent the space of evolved sequences as they are subject to acquisition bias towards available species of interest. To account for uneven representation, sequence reweighting has been introduced to lower the contributions of highly similar sequences and assign higher weight to unique ones (see Durbin et al. [44], p. 124 ff.). In particular, the weight of the *m*-th sequence, *w*_*m*_: = 1/*k*_*m*_, in the alignment {**x** ^1^,…, **x** ^*M*^}, can be chosen to be the inverse of $k_{m}:=\sum_{n=1}^{M}H\left(\sum_{i=1}^{L}1(x_{i}^{m},x_{i}^{n})-L\cdot \theta \right)$, the number of sequences **x** ^*m*^ shares more than *θ* · 100% of its residues with. Here, *θ* denotes a similarity threshold and is typically chosen as 0.7 ≤ *θ* ≤ 0.9, **1**(*a*,*b*) = 1 if *a* = *b* and **1**(*a*,*b*) = 0, otherwise, and *H* is the step function with *H*(*y*) = 0 if *y* < 0 and *H*(*y*) = 1, otherwise. This also provides us with an estimate of the effective number of sequences in the alignment,$M_{\text{eff}}:=\sum_{m=1}^{M}w_{m}$. Additionally, pseudocount regularization with $\tilde{\lambda }>0$ is used to deal with finite sampling bias and to account for underrepresentation [5–8,44,48], resulting in zero entries in $\hat{C}(\sigma ,\omega )$, for instance, if a certain amino acid pair is never observed. The use of pseudocounts is equivalent to a maximum a posteriori (MAP) estimate under a specific inverse Wishart prior on the covariance matrix [48]. Both preprocessing steps combined yield the reweighted single and pair frequency counts,

$$f_{i}(\sigma )=\frac{1}{M_{\text{eff}}+\tilde{\lambda }}\left(\frac{\tilde{\lambda }}{q}+\sum_{m=1}^{M}w_{m}x_{i}^{m}(\sigma )\right),\;f_{ij}(\sigma ,\omega )=\frac{1}{M_{\text{eff}}+\tilde{\lambda }}\left(\frac{\tilde{\lambda }}{q^{2}}+\sum_{m=1}^{M}w_{m}x_{i}^{m}(\sigma )x_{j}^{m}(\omega )\right),$$

in residues *i*,*j* = 1,…, *L* and for amino acids *σ*,*ω*∈Ω. Ideally for maximum-likelihood inference, the random variables are assumed to be independent and identically distributed. However, this is typically violated in realistic sequence data due to phylogenetic and sequencing bias, and the reweighting presented here does not necessarily solve this problem.

## Scoring Functions for the Pairwise Interaction Strengths

For pairwise maximum-entropy models of continuous variables, the natural scoring function for the interaction strength between two variables *x*_*i*_ and *x*_*j*_, given the inferred inverse covariance matrix, is the partial correlation Eq 18. However, for categorical variables, the situation is more complicated, and there are several alternative choices of scoring functions. Requirements on the scoring function are that it has to account for the chosen gauge and, in the case of protein contact prediction, evaluate the coupling strength between two residues *i* and *j* summarized across all possible *q* ^2^ amino acids pairs. The highest scoring residue pair is, for instance, used to predict the 3-D structure of the protein of interest. For this purpose, the direct information, defined as the mutual information applied to $P_{ij}^{\text{dir}}(\sigma ,\omega )=\frac{1}{Z_{ij}}\text{exp}\left(e_{ij}(\sigma ,\omega )+{\tilde{h}}_{i}(\sigma )+{\tilde{h}}_{j}(\omega )\right)$ instead of *f*_*ij*_(*σ*,*ω*),

$${\text{DI}}_{ij}=\sum_{\sigma ,\omega \in \Omega }P_{ij}^{\text{dir}}(\sigma ,\omega )\text{ln}\left(\frac{P_{ij}^{\text{dir}}(\sigma ,\omega )}{f_{i}(\sigma )f_{j}(\omega )}\right),$$

has been introduced [5]. In $P_{ij}^{\text{dir}}(\sigma ,\omega )$, ${\tilde{h}}_{i}(\sigma )$ and ${\tilde{h}}_{j}(\omega )$ are chosen to be consistent with the (reweighted and regularized) single-site frequency counts, *f*_*i*_(*σ*) and *f*_*j*_(*ω*), and *Z*_*ij*_ such that the sum over all pairs (*i*, *j*) with 1 ≤ *i* < *j* ≤ *L* is normalized to 1. The direct information is invariant under gauge changes of the Hamiltonian ℋ, which means that any suitable gauge choice results in the same scoring values. As an alternative measure of the interaction strength for a particular pair (*i*, *j*), the Frobenius norm of the 21×21-submatrices of (*e*_*ij*_(*σ*,*ω*))_σ,ω_ has been used,

$$∥e_{ij}{∥}_{\text{F}}={\left(\sum_{\sigma ,\omega \in \Omega }e_{ij}{\left(\sigma ,\omega \right)}^{2}\right)}^{1/2}.$$

However, this expression is not gauge-invariant [5]. In this context, the notation with *e*_*ij*_(*σ*,*ω*), which refers to indices restricted to *i* < *j*, is extended and treated such that *e*_*ij*_(*σ*,*ω*) = *e*_*ji*_(*ω*,*σ*) and *e*_*ij*_(·,·) = 0; then ||*e*_*ij*_||_F_ = ||*e*_*ji*_||_F_ and ||*e*_*ii*_||_F_ = 0. In order to correct for phylogenetic biases in the identification of co-evolved residues, Dunn et al. [27] introduced the average product correction (APC). It has been originally used in combination with the mutual information but was recently combined with the *ℓ* ^1^-norm [8] and the Frobenius/*ℓ* ^2^-norm [51] and is derived from the averages over rows and columns of the corresponding norm of the matrix of the *e*_*ij*_ parameters. In this formulation, the pair scoring function is

$${\text{APC-FN}}_{ij}=∥e_{ij}{∥}_{\text{F}}-\frac{∥e_{i\cdot }{∥}_{\text{F}}∥e_{\cdot j}{∥}_{\text{F}}}{∥e_{\cdot \cdot }{∥}_{\text{F}}}$$ (24)

for *e*_*ij*_-parameters fixed by zero-sum gauge and with the means over the non-zero elements in row, column and full matrix, $∥e_{i\cdot }{∥}_{\text{F}}:=\frac{1}{L-1}\sum_{j=1}^{L}∥e_{ij}{∥}_{\text{F}}$, $∥e_{\cdot j}{∥}_{\text{F}}:=\frac{1}{L-1}\sum_{i=1}^{L}∥e_{ij}{∥}_{\text{F}}$ and $∥e_{\cdot \cdot }{∥}_{\text{F}}:=\frac{1}{L(L-1)}\sum_{i,j=1}^{L}∥e_{ij}{∥}_{\text{F}}$, respectively. Alternatively, the average product-corrected *ℓ* ^1^-norm applied to the 20×20-submatrices of the estimated inverse covariance matrix, in which contributions from gaps are ignored, has been introduced by the authors of [8] as the Psicov-score. Using the average product correction, the authors of [51] showed for interaction parameters inferred by the mean-field approximation that scoring with the average product-corrected Frobenius norm increased the precision of the predicted contacts compared to scoring with the DI-score. The practical consequence of the choice of scoring method depends on the dataset and the parameter inference method.

## Discussion of Results, Improvements, and Applications

**Fig 3:**
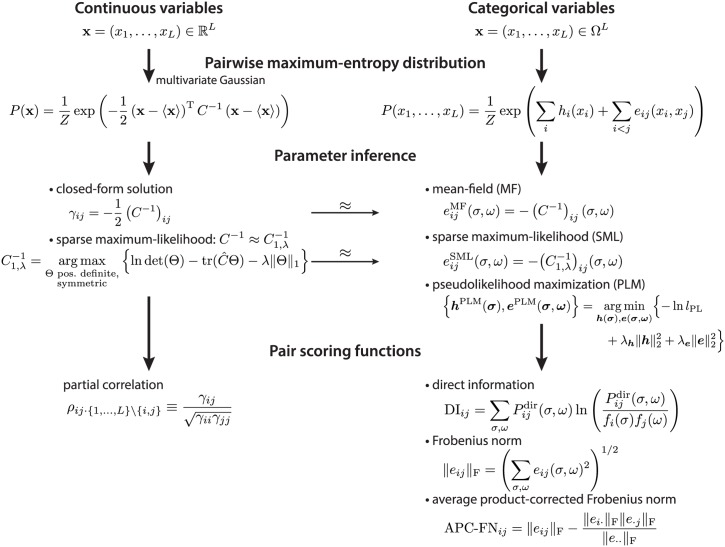
Scheme of pairwise maximum-entropy probability models. The maximum-entropy probability distribution with pairwise constraints for continuous random variables is the multivariate Gaussian distribution (left column). For the maximum-entropy probability distribution in the categorical variable case (right column), various approximative solutions exist, e.g., the mean-field, the sparse maximum-likelihood, and the pseudolikelihood maximization solution. The mean-field and the sparse maximum-likelihood result can be derived from the Gaussian approximation of binarized categorical variables (thin arrow). Pair scoring functions for the continuous case are the partial correlations (left column). For the categorical variable case, the direct information, the Frobenius norm, and the average product-corrected Frobenius norm are used to score pair couplings from the inferred parameters (right column).

Maximum entropy-based inference methods can help in estimating interactions underlying biological data. This class of models, combined with suitable methods for inferring their numerical parameters, has been shown to reveal—to a reasonable approximation—the direct interactions in many biological applications, such as gene expression or protein residue—residue coevolution studies. In this review, we have presented maximum-entropy models for the continuous and categorical random variable case. Both approaches can be integrated into a framework, which allows the use of solutions obtained for continuous variables as approximations for the categorical random variable case (Fig 3).

The validity and precision of the available maximum-entropy methods could be improved to yield more biologically insightful results in several ways. Advanced approximation methods derived from Ising model approaches [59,71] are possible extensions for efficient inference. Moreover, additional terms beyond pair interactions can be included in models of continuous and discrete random variables [1,33,59]. However, higher-order models demand more data, which is a major bottleneck for their application to biological problems. In the case of protein contact prediction, this could be resolved by getting more sequences, which are being obtained as the result of extraordinary advances in sequencing technology. The quality of existing methods can be improved by careful refinement of sequence alignments in terms of cutoffs and gaps or by attaching optimized weights to each of the data sequences. Alternatively, one could try to improve the existing model frameworks by accounting for phylogenetic progression [27,49,72] and finite sampling biases.

The advancement of inference methods for biological datasets could help solve many interesting biological problems, such as protein design or the analysis of multi-gene effects in relating variants to phenotypic changes as well as multi-genic traits [73,74]. The methods presented here could help reduce the parameter space of genome-wide association studies to first approximation. In particular, we envision the following applications: (1) in the disease context, co-evolution studies of oncogenic events, for example copy number alterations, mutations, fusions and alternative splicing, can be used to derive direct co-evolution signatures of cancer from available data, such as The Cancer Genome Atlas (TCGA); (2) *de novo* design of protein sequences as, for example, described in [65,75] for the WW domain using design rules based on the evolutionary information extracted from the multiple sequence alignment; and (3) develop quantitative models of protein fitness computed from sequence information.

In general, in a complex biological system, it is often useful for descriptive and predictive purposes to derive the interactions that define the properties of the system. With the methods presented here and available software (Table 1), our goal is not only to describe how to infer these interactions but also to highlight tools for the prediction and redesign of properties of biological systems.

**Table 1:** Overview of software tools to infer pairwise interactions from datasets in continuous or categorical variables with maximum-entropy/GGM-based methods.

Data type	Method	Name	Output	Link
categorical	mean-field	DCA, mfDCA	DI-score	[76,77]
	pseudolikelihood maximization	plmDCA	APC-FN-score	[78–80]
	pseudolikelihood maximization	Gremlin	Gremlin-score	[81]
	sparse maximum-likelihood	Psicov	Psicov-score	[82]
continuous	sparse maximum-likelihood	glasso	partial correlations	[83]
	*ℓ* ^2^-regularized maximum-likelihood	scout	partial correlations	[84]
	shrinkage	corpcor, GeneNet	partial correlations	[85,86]
